# Courting disaster: How diversification rate affects fitness under risk

**DOI:** 10.1111/evo.12568

**Published:** 2014-12-23

**Authors:** William C Ratcliff, Peter Hawthorne, Eric Libby

**Affiliations:** 1 School of Biology, Georgia Institute of TechnologyAtlanta, Georgia 30332; 3 Applied Economics, University of MinnesotaSt. Paul, Minnesota 55104; 4 Santa Fe InstituteSanta Fe, New Mexico 87501

**Keywords:** Bistability, environmental uncertainty, life-history evolution, metapopulation modeling, stochastic switching

## Abstract

Life is full of risk. To deal with this uncertainty, many organisms have evolved bet-hedging strategies that spread risk through phenotypic diversification. These rates of diversification can vary by orders of magnitude in different species. Here we examine how key characteristics of risk and organismal ecology affect the fitness consequences of variation in diversification rate. We find that rapid diversification is strongly favored when the risk faced has a wide spatial extent, with a single disaster affecting a large fraction of the population. This advantage is especially great in small populations subject to frequent disaster. In contrast, when risk is correlated through time, slow diversification is favored because it allows adaptive tracking of disasters that tend to occur in series. Naturally evolved diversification mechanisms in diverse organisms facing a broad array of environmental risks largely support these results. The theory presented in this article provides a testable ecological hypothesis to explain the prevalence of slow stochastic switching among microbes and rapid, within-clutch diversification strategies among plants and animals.

All organisms face the possibility of environmental change. Such variation can be costly, reducing fitness or leading to the extinction of previously well-adapted organisms (Lande and Orzack 1988). When change is predictable or sufficiently gradual, these negative effects can be ameliorated by adaptive phenotypic plasticity (Via and Lande 1985; Thompson 1991). Alternatively, organisms may respond to change by adaptively tracking their environment over generations (Burger and Lynch 1995). These modes of response may not be effective when environmental change is severe and unpredictable, as is the case for many periodic stresses. Under these conditions, risk avoidance through “bet hedging” can increase fitness (Levins 1962; Cohen 1966; Bull 1987; Clauss and Venable 2000; Meyers and Bull 2002; Donaldson-Matasci et al. 2008; Simons 2009).

The theory of bet hedging, developed originally by physicist Daniel Bernoulli in 1738 to aid human decision making (Stearns 2000), is based on the premise that Darwinian fitness, much like an investor's returns, is best described by the long-term average of a multiplicative series (a geometric mean). Because the geometric mean is sensitive to variance, long-term fitness can be improved by bet hedging: either diversifying or adopting a conservative phenotype that reduces across-generation variance in the face of uncertainty. In this article we focus on diversification bet hedging, a widely employed adaptation to unpredictable environmental change that has evolved in taxonomically divergent lineages (Simons 2011).

Diversification rates vary dramatically among different organisms. Macroorganisms (metazoans, plants, and multicellular fungi) tend to diversify by producing heterogeneous offspring each time they reproduce. For example, when the optimal timing of plant seed germination varies among years, selection favors diversification for this trait (Simons 2009). Individual seed-producing plants impose dormancy of varying duration on their seeds, resulting in the production of offspring, which follow a distribution of dormancy that maximizes the seed-producing parent's geometric mean fitness (Simons 2009). Within-clutch phenotypic variability, consistent with diversification bet hedging, has been observed in diverse organisms, ranging from microbes (Ratcliff and Denison 2010) to metazoans (Hopper 1999; Lips 2001; Laaksonen 2004; Crean and Marshall 2009; Simons 2011).

In contrast, microbes tend to diversify through stochastic phenotype switching (Andrewes 1922; Balaban et al. 2004; Beaumont et al. 2009). Individuals produce offspring that are phenotypically similar to the parent, but have a low probability of producing offspring with an alternative phenotype (ranging from 10^−1^ to 10^−5^ per offspring, with 10^−3^ being a typical rate) (van der Woude and Baumler 2004). This new phenotype is inherited, and future offspring can stochastically revert to the ancestral phenotype. Individuals rarely produce heterogeneous offspring, but diversity can be created among clonemates when the population is composed of multiple genetically identical, but phenotypically heterogeneous, lineages (Fig. S1a). Stochastic diversification can be relatively slow: at a typical switch rate of 10^−3^/generation, it takes more than 1000 generations to generate maximal diversity (Fig. S1b). Stochastic diversification in microbes results from a number of well-studied molecular mechanisms. For example, phase variation and contingency loci result in high-frequency genetic changes that turn on or off individual genes or operons (van der Woude and Baumler 2004; Moxon et al. 2006). Alternatively, a positive feedback loop in gene expression can result in bistable phenotypic differentiation (with an activated “on” subpopulation, and a deactivated “off” subpopulation; Gordon et al. 2009). So far, stochastic phenotype switching has only been described in microbes.

In this article, we examine how key attributes of risk and organismal ecology affect the fitness consequences of different diversification rates. Specifically, we consider the spatial and temporal dynamics of uncertainty. A single unpredictable event may vary in scale from population-wide (e.g., a landscape-level process like unpredictable season length) to local (e.g., chance of nest discovery by a predator). Similarly, risk may affect populations randomly in time or it may occur in correlated series. Using the above examples, season length is largely uncorrelated from year to year, but a predator that discovers a nest site may revisit it frequently. We find that the fitness effects of diversification depend heavily on the structure of risk. Rapid diversification (the faster the better) is favored when risk has a large spatial extent, whereas slower rates of diversification are favored when risk is correlated in time. We also examine the effects of population size, disaster frequency, and among-patch migration (both rate and distance) on the fitness consequences of slow versus fast diversification. Finally, we investigate how different organisms have evolved to hedge against divergent forms of risk, and use our results to explain why microorganisms tend to evolve slow, stochastic differentiation strategies, whereas macroorganisms tend to diversify every generation.

## Methods and Results

We consider a population of sequentially reproducing organisms divided into subpopulations that occupy different patches. Each organism exists in one of two phenotypic states, A or B. The particular phenotype does not affect any fitness parameters except susceptibility to risk. To compare fast versus slow diversification rates, we populate our simulation with two organismal genotypes that differ only in the frequency of switching between A and B phenotypes. One genotype, *D_f_*, is a fast diversifier and switches between A and B upon every cell division. The other, *D_s_*, is a slow diversifier and produces offspring of a different phenotype (A cells produce B cells, and vice versa) with probability *P*, where *P* < 1 (see Fig. S1 for a schematic). Similar to the molecular mechanism of phase variation, we assume that an individual's phenotype is determined at birth and does not change during their lifetime.

We discretize time so that at each time step, there is a probability *k* of disaster striking and killing either all A or B phenotypes in a fixed number of randomly chosen patches. This can be thought of as an environmental catastrophe (e.g., recognition by an immune system, cold weather, or antibiotic exposure) to which only one phenotype is resistant. Following implementation of disasters, organisms in all patches undergo population turnover with a round of death and reproduction. Each organism experiences a probability α of dying from factors unrelated to disasters. Afterwards each patch is restored to its carrying capacity via population growth. Organisms are sampled at random from each patch and allowed to produce an offspring until the carrying capacity is reached. Every simulation starts with all patches filled randomly with the two organisms and their A and B phenotypes (*D_f_* phenotype A, *D_f_* phenotype B, *D_s_* phenotype A, and *D_s_* phenotype B).

In our model, the first disaster has an equal chance of killing either A or B phenotype individuals. When disaster is uncorrelated in time, then the probability that the next disaster kills A or B phenotype individuals cannot be predicted from the last disaster. In this case, a 50:50 ratio of A:B phenotypes ensures the lowest among-patch variance in mortality during disaster, and thus the highest geometric mean fitness (Cohen 1966). *D_f_* generates this phenotypic ratio within a single generation, making it unbeatable by the slower process of stochastic diversification. Rapid diversification may not always be adaptive, however. If a type of disaster (e.g., killing A or B cells) tends to repeat through time, then slower diversification may be advantageous, because it can result in production of offspring with phenotypes well-suited to upcoming disasters (see below for a full explanation). Using our metapopulation simulation model, we examine the effect of risk structure and other key ecological factors on the fitness consequences of diversification rate.

### Ecological Drivers of Fitness Differences Between Diversification Strategies

#### Population size and frequency of disaster

**Figure 1:**
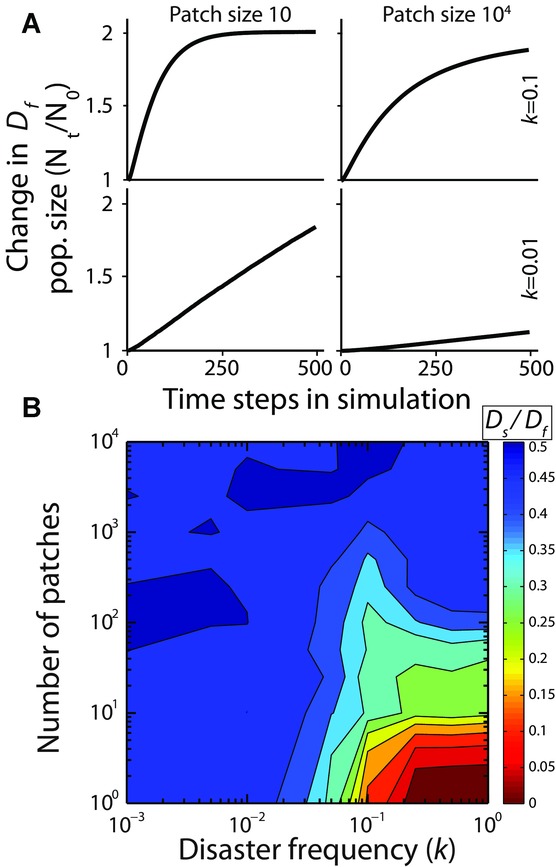
Population size and the spatial extent of risk. (A) Slow diversification is less effective in small patches subject to frequent disaster. *D_f_* displaces *D_s_* more rapidly when the carrying capacity of each patch is only 10 individuals, compared with 10,000, and when disaster (*k*) strikes with a higher probability. Small patch size is problematic for the slow diversifier, as their chance of producing diversified offspring via stochastic switching is reduced when there are fewer individuals in the group. For these simulations, *a* = 0.1, *m* = 0, *P* = 0.001. (B) Rapid diversification is especially favored by natural selection when risk is frequent (greater values of *k*) and each disaster affects a large fraction of the population (organisms distributed across a small number of patches). Competitions between fast and slow switchers were run for 50 time steps (repeated for 100 simulations in which neither strain went extinct). In both (A) and (B), the metapopulation was seeded with equal frequencies of *D_s_* and *D_f_*. The z-scale represents the ratio (which started at 0.5) of *D_s_* and *D_f_* after 50 time steps.

In 1000-patch metapopulations, we simulated conditions where the carrying capacity of each patch was either small (10 individuals) or large (10^4^ individuals), resulting in maximum global population sizes of 10^4^ or 10^7^ individuals, respectively. Both population sizes were simulated with high (*k* = 0.1) and low (*k* = 0.01) frequencies of disaster. All four combinations of parameters favored rapid diversification. This is expected because there is no cost to diversifying quickly, but there is a cost to failing to diversify. Although all conditions favor rapid diversification, they do so at different rates. Smaller populations subject to more frequent disaster provides the strongest selection for rapid diversification (Fig. 1A). This is because both reduce *D_s_*'s chance of diversifying before the next disaster strikes, leading to a higher probability of local extinction.

#### Spatial extent of risk

One critical (but often overlooked) aspect of risk is its spatial and temporal scale. Specifically, a single risk factor can range in effect from a very small spatial scale, affecting only a small subset of the population, to the landscape level, affecting the entire population. Further, the type of disaster that occurs (i.e., killing A or B phenotype individuals) can be independent with respect to time, or may tend to recur in the same manner repeatedly. We refer to these as the *spatial extent of risk* and the *temporal autocorrelation of risk*, respectively. Spatial extent is determined by the fraction of the individuals in the population that experience the same disaster, whereas temporal autocorrelation can be determined by the probability that the next disaster to strike a patch is the same as the last disaster that affected the patch.

To examine how the spatial extent of risk affects diversification strategies, we simulated a metapopulation in which the global population size was kept fixed at 10^6^ organisms but varied the number of patches that independently experienced disasters. At one end of the spectrum there is a single patch that contains the entire population, as a result all individuals experience the exact same risk (high spatial extent). At the other end of the spectrum the population is subdivided among 10,000 patches, each with small populations experiencing disaster independently from each other (low spatial extent of risk). Simulations of these different spatial structures show that rapid diversification is most favored when risk affects a broad subset of the population (high spatial extent, Fig. 1B). Lowering the spatial extent of risk reduces the cost of slow diversification because different subsets of the population independently experience disaster, and therefore the genotype as a whole experiences less variation in fitness.

#### Temporal autocorrelation of risk

To examine how temporal autocorrelation affects diversification strategies, we repeated the above simulation, but with a critical difference: the first disaster to strike a patch was still random (killing either A or B phenotype individuals), but each subsequent disaster to strike that patch killed the same phenotype as the previous disaster with a 90% probability.

**Figure 2:**
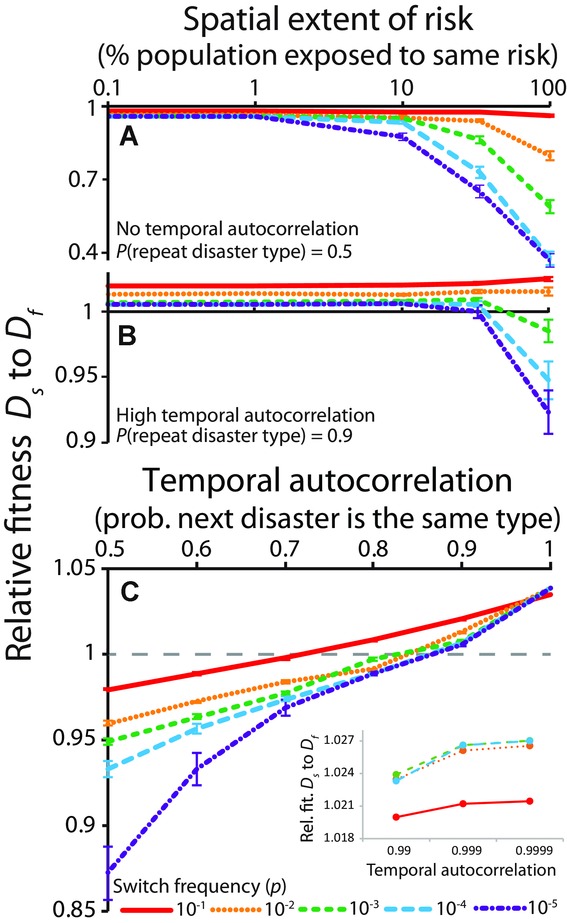
Effect of spatial extent and temporal autocorrelation of risk on the relative success of each diversification strategy. (A) When there is no tendency for the type of disaster to repeat through time, faster diversification is always favored by natural selection. The fitness difference between fast and slow strategies is greatest when a single event affects a larger fraction of the population. (B) Rapid diversification can be costly when risk is temporally autocorrelated, however, because it creates many offspring that will likely succumb to the next disaster. (C) Slower rates of diversification are thus evolutionarily favored when a patch is likely to experience the same disaster type repeatedly. Dashed gray line in (C) demarcates equivalent fitness. Inset examines high temporal autocorrelations. Error bars are 95% confidence intervals from 1000 simulations. For all simulations, *a* = 0.1, *k* = 0.1, *m* = 0, overall population size was 10^6^. Patch number was varied from 1 to 1000 in (A, B) and was 10 in (C). Relative fitness is calculated as the ratio of the geometric mean change in population size for each strain over 50 time steps: 
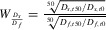
.

For the following simulations, we fix the rate of disaster at *k* = 0.1, because the fitness consequences of diversification rate depend strongly on the spatial extent of risk at this disaster interval (Fig. 1B). When risk is characterized by high temporal autocorrelation, intermediate diversification rates were favored (Fig. 2B, C). In fact, *D_f_* only had an advantage in the conditions where it was most heavily favored before, namely very high spatial extent of risk and very low switching frequencies by *D_s_*. Risk that is more temporally autocorrelated favors slower diversification rates (Fig. 2C, inset).

#### Migration

**Figure 3:**
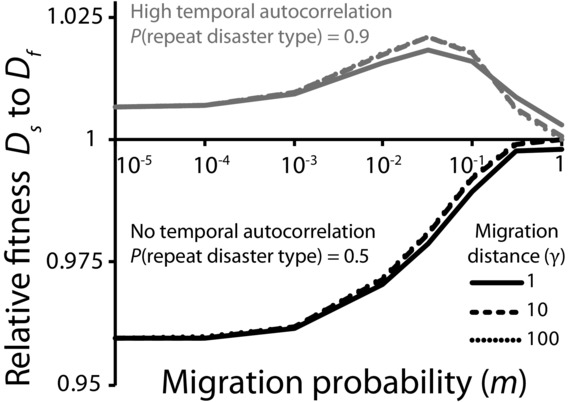
The effect of migration on *D_s_*'s relative fitness depends on whether the specific type of disaster is likely to repeat. When there is no temporal autocorrelation of risk, migration reduces the correlation between units of selection and risk, decreasing the effect of phenotypic diversification on fitness. As a result, increased migration reduces *D_f_*'s fitness advantage. In contrast, when a specific type of disaster is likely to repeat, intermediate migration rates maximize *D_s_*'s relative advantage. All 95% confidence intervals ≤10^−4^ and were not drawn. For all simulations, *a* = 0.1, *k* = 0.1, *P* = 0.001, with 1000 patches in the metapopulation with an overall carrying capacity of 10^6^ individuals. Relative fitness is calculated as the ratio of geometric mean changes in population size over 50 time steps.

To understand the effects of migration, we impose a structure on the patches and add a step to our model. We use a simple one-dimensional ring structure (i.e., a linear array with periodic boundary conditions) in which every patch is connected to two other patches. After disaster strikes a patch, each surviving individual has a probability *m* of migrating to a new, randomly selected patch within distance γ. Migration between patches tends to break up the correlation between units of selection and risk. This is because risk is applied separately to different subpopulations, so the more a lineage spreads across multiple subpopulations (even if it is phenotypically homogeneous), the more effectively it is diversified relative to risk. When risk is not temporally autocorrelated, higher rates of migration monotonically reduce the difference in fitness between *D_f_* and *D_s_* (Fig. 3). However, when there is strong temporal autocorrelation [*P*(disaster type is same) = 0.9], intermediate rates of migration increase *D_s_*'s relative fitness (Fig. 3). This is due to a trade-off imposed by slow diversification under temporally autocorrelated risk: producing self-similar offspring that will likely survive the next disaster increases fitness on average, but exposes the poorly diversified genotype to a risk of high mortality if the disaster type changes. Migration mitigates this cost, allowing some individuals to move to new patches with decoupled risk. Regardless of temporal autocorrelation, migration distance had little effect.

#### Uni- versus multicellular life histories

Our simulation model considers an organism that reproduces sequentially, like a bacterium. We modeled this type of reproduction (rather than a multicellular life history in which reproduction occurs through multi-offspring clutches) because it is simpler, clearly illustrating the key effects of variation in diversification rate on fitness. To determine if our results are sensitive to reproductive mode, we modified our model to consider competition between multicellular organisms that reproduce through multi-offspring clutches. Our main conclusions were not affected by whether the focal organism has a unicellular or multicellular life history (Fig. S2). Specifically, even a macroorganism capable of producing multi-offspring clutches benefits from slow, stochastic diversification when risk is temporally autocorrelated.

### Bet Hedging in the Real World

**Figure 4:**
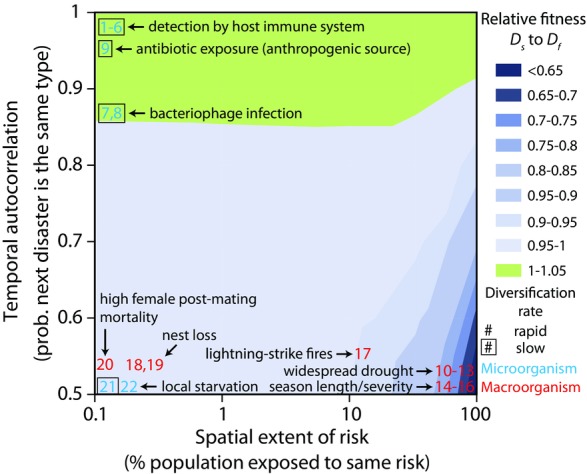
Bet hedging under diverse risk structures. High temporal autocorrelation, but low spatial extent of risk favors slow rates of diversification. This can occur if the phenotype of the diversifying organism affects the type of risk it experiences (examples 1–9), such as recognition of a specific bacterial antigen by an adaptive immune system. In contrast, rapid diversification is favored when risk is not temporally autocorrelated, and any disaster affects a large fraction of the population (examples 10–17). Rapid diversification is slightly favored for the remainder of the state space, including when the spatial extent and temporal autocorrelation of risk are low (examples 18–22). To generate the phase diagram, we simulated 1000 runs of a metapopulation with an overall carrying capacity of 10^6^ individuals in which patch number was varied from 1 to 1000. *a* = 0.1, *k* = 0.1, *m* = 0, *P* = 0.001. Relative fitness is calculated as the ratio of geometric mean changes in population size over 50 time steps.

Our analyses demonstrate that the relative success of different diversification rates depends largely on the spatial extent and temporal autocorrelation of risk (Fig. 2). We compare these factors directly in Figure 4. Here we find that instantaneous diversification is strongly favored when the spatial extent of risk is high and temporal autocorrelation is low, whereas slower stochastic diversification (*P* = 0.001) is favored only when temporal autocorrelation is high. How do naturally evolved bet-hedging mechanisms fit this pattern? Unfortunately, data on the frequency and extent of disaster in nature are scarce (this is an endemic problem in the field of bet hedging; Hopper 1999; Simons 2011). As a result, we estimate the spatial extent and temporal autocorrelation of risk based on the type of risk experienced for 22 published examples of diversification bet hedging. In all of the macroorganismal examples, individuals produce well-diversified offspring in a single generation. We refer to this as “within-clutch” diversity, even if offspring are not produced in physical clutches. The physical aggregation of offspring is irrelevant with respect to our model. What matters is that diversified offspring are produced rapidly. In contrast, our microbial examples display much more variation in diversification rate. The *x*-*y* coordinates of each example (demarcated by the superscript numbers in the following paragraphs, which correspond to the numbers superimposed over Fig. 4) should not be considered a datapoint, but rather a hypothesis. It is our hope that, collectively, these hypotheses provide heuristic insight into how the spatial extent and temporal autocorrelation of risk may affect the type of diversification that evolves.

Several broad categories of risk can be identified: the first type is characterized by unpredictable initial disaster, followed by disasters of the same type (high temporal autocorrelation). One way this can occur is if the agent causing the disaster interacts with the organisms that are diversifying. For example, some pathogens create antigenic diversity: (Gjini et al. 2010), (Hagblom et al. 1985), (Zhang et al. 1997), (Skare et al. 1995), (McKevitt et al. 2005), and (Keely et al. 2005). Once an antigen is detected by an adaptive immune system, that phenotype will continue to be killed, creating high temporal autocorrelation. Similarly, bacteriophage capable of infecting only one of several bacterial phenotypes (Zaleski et al. 2005; Labrie et al. 2010) may persist in the local environment, infecting any subsequent susceptible bacteria produced in that area. Other risks simply come in series. For example, pathogenic *Pseudomonas* create phenotypic diversity for antibiotic susceptibility in cystic fibrosis patients (Drenkard and Ausubel 2002). Treatment of infection often consists of repeated doses of the same drug (Frederiksen et al. 1997), generating high temporal autocorrelation. Slow diversification is evolutionarily favored in this category, and organisms facing this risk tend to create diversity by low-frequency stochastic switching (Fig. 4, upper left).

A second major category of risk is landscape-level phenomena that affect most or all of the population (high spatial extent). For example, some plants produce both dormant and nondormant seeds in the face of unpredictable, widespread drought: (Clauss and Venable 2000), (Danforth 1999), (Evans and Dennehy 2005), (Philippi 1993). Although it might appear that there is only one “disaster type” here (drought), functionally there are two: dormant seeds survive during drought years, but pay a severe opportunity cost during wet years (Cohen 1966). So from a dormant seed's perspective, a wet year is a disaster, and a series of either wet or dry years would cause high variance in fitness. Where environmental stress only kills a single phenotype, and there are fitness trade-offs between phenotypes suited to stress and nonstress conditions (such as drought), we estimate the temporal autocorrelation of stress and nonstress conditions. Like widespread drought, variation in season length (Bradford and Roff 1993; Groeters 1994) and severity (Arthur et al. 1973) tend to affect the entire population. These are paradigmatic examples of low temporal autocorrelation, because disaster is caused by climatological factors that in general are not dependent on the prior year's weather. We also observe examples of intermediate spatial extent of risk, such as *Pityopsis graminifolia*: faced with frequent lightning strike induced fires that kill plants in coarse patches, this species has evolved to produce offspring that either depend on fire for flowering, or flower independently (Brewer 2008). Rapid diversification is strongly favored in these conditions, and organisms facing risk with a large spatial extent tend to diversify every generation (Fig. 4, lower right).

Finally, some risks are characterized by both low spatial extent and low temporal autocorrelation. Examples here are *Pseudophryne* frogs (Byrne and Keogh 2009) that lay their eggs in multiple nest sites, and *Aedes* mosquitoes that produce eggs that hatch asynchronously (Khatchikian et al. 2010), increasing the chance that some offspring survive temporary nest-site desiccation. In response to high female postmating mortality, dasyurid marsupials have evolved male semelparity and polygyny (Kraaijeveld et al. 2003). Microbes too fall into this category: *Bacillus* diversify into swimming and sessile phenotypes that reduce the risk of local starvation (Chai et al. 2010), whereas *Sinorhizobium* hedge against local starvation by asymmetrically provisioning resources during division (Ratcliff and Denison 2010). Diversification rate has little effect on fitness when the spatial extent and temporal autocorrelation of risk are low. Indeed, we see both rapid (18–20, 22) and slow (21) diversification strategies in this category (Fig. 4, lower left).

## Discussion

Unpredictable environmental change poses a fundamental challenge for natural selection. One way that organisms can adapt to variable environments is through adaptive phenotypic diversification, a process known as bet hedging. Although many different taxa have evolved to bet hedge (Simons 2011), their diversification strategies are not all equivalent. In this article, we show that fast and slow diversification strategies are uniquely suited to hedging against different forms of risk. Rapid diversification is evolutionarily favored when risk is not temporally autocorrelated, because it increases the likelihood that an optimal level of diversity will be generated before the next disaster. This effect is magnified when the risk faced is characterized by high spatial extent (lower right corner of Fig. 4), because across-generation variation in fitness is greatest when a single disaster affects the entire population (Hopper et al. 2003; Starrfelt and Kokko 2012). In contrast, slow diversification rates are favored when risk is correlated in time (top of Fig. 4). Slower diversification is beneficial when the same type of disaster tends to reoccur, because most offspring will possess the phenotype of their (recently successful) parents, which is likely to be suited to the next disaster. Thus, slow diversification allows for phenotypic tracking of disasters that tend to occur in series.

A review of the bet-hedging literature reveals a trend broadly consistent with our model: rapid, within-clutch diversification has evolved under conditions that strongly favor it (i.e., high spatial extent but low temporal autocorrelation), whereas slow, stochastic diversification has evolved under conditions favoring it (i.e., high temporal autocorrelation). Our literature review is not intended to be comprehensive, instead we choose examples that were representative of different types of risk. Importantly, we were unable to find any examples of bet hedging that deviate from this general pattern (i.e., no examples of rapid, within-clutch diversification for risks characterized by high temporal autocorrelation, or slow stochastic diversification for risks characterized by high spatial extent, but low temporal autocorrelation).

Although the spatial and temporal characteristics of risk determine which bet-hedging strategy is favored, other key ecological factors affect the magnitude of the difference between strategies. Slow stochastic diversification is less effective in smaller populations. After selection removes one phenotype from a patch, the length of time required for the first phenotypic variants to be produced declines with increased population size. Further, because stochastic phenotype switching is subject to sampling error, smaller populations experience more variance in diversity. This variance is costly, as any deviation from the optimal phenotype ratio reduces geometric mean fitness. Similarly, more frequent disasters (with respect to the number of intervening generations) reduce the relative fitness of slow diversifiers, because disaster will often occur before a high level of diversity has been generated. High rates of migration among patches spread risk in a manner similar to phenotypic diversification (Starrfelt and Kokko 2012), which has contrasting effects depending on temporal autocorrelation. When risk is temporally autocorrelated, intermediate rates of migration increase the fitness of slow relative to fast diversifiers, because this allows them to mitigate the high mortality that occurs when disaster type switches. When risk is not temporally autocorrelated, migration reduces the importance of bet hedging to fitness, decreasing the difference in fitness between fast and slow diversifiers.

These results raise a key question: why do microorganisms tend to pursue slow, stochastic diversification strategies, whereas macroorganisms produce well-diversified offspring each time they reproduce? This pattern is not one created by physiological constraints: microbes possess the ability to deterministically produce offspring that are phenotypically different from the parent cell (Colman-Lerner et al. 2001; Lindner et al. 2008; Ratcliff and Denison 2010; Levy et al. 2012), and stochastic processes can play a fundamental role in multicellular development (Riedel-Kruse et al. 2007; Cohen et al. 2010). We hypothesize this observation is due to both ecological differences between micro- and macroorganisms, and publication bias. Relative to multicellular organisms, microbes tend to have enormous population sizes, patchy spatial distributions (reducing the spatial extent of risk), and a rapid rate of generational turnover (McArthur 2006). All of these traits increase the efficacy of slow, stochastic diversification. Further, microbes may simply face more risks for which slow diversification is optimal (e.g., temporally autocorrelated disasters such as immune system recognition or repeated antibiotic exposure).

Caution, however, is in order when drawing large-scale conclusions from the literature on bet hedging, as substantial publication biases exist. The microbial bet-hedging literature is dominated by stochastic switching to the point that “stochastic switching” and “bet hedging” are often used synonymously. Stochastic switching may be oversampled, relative to deterministic diversification, largely because microbial ecology is more challenging to study than microbial cell biology. The mechanistic bases of stochastic phenotype switching (e.g., phase variable genes, contingency loci, and positive-feedback loops in gene expression) have been exactingly described (van der Woude and Baumler 2004; Moxon et al. 2006; Bayliss 2009; Gordon et al. 2009), and are widely assumed to be adaptations resulting from natural selection. The argument that stochastic switching has evolved *for the purpose* of diversification has therefore been seen as compelling, even in the absence of evidence that stochastic switching increases geometric mean fitness in natural conditions. Because of the difficulties of studying most microbes in their natural environment, it has been difficult to link other sources of among-cell variation (e.g., phenotypic differences between new- and old-pole cells [Lindner et al. 2008] or daughter cell specific gene expression [Colman-Lerner et al. 2001]) to fitness. Further work examining less conventional diversification mechanisms may yield novel microbial bet-hedging strategies that act to rapidly create diversity.

In this article, we model a microbe with stochastic phenotype switching. This naturally raises a question of relevance to macro-organisms that bet hedge by producing clutches of diversified offspring. In general, our main conclusions should apply to both micro- and macroorganisms: producing well-diversified offspring is adaptive if risk is not correlated in time, while producing mainly self-similar offspring is beneficial under temporally autocorrelated risk. An organism's ability to express adaptive diversification regimes will likely depend on how within-clutch diversity is created. If offspring in a clutch inherit their parent's phenotype with a fixed probability, then our results stand unchanged (Fig. S2). Offspring phenotype may also be assigned deterministically. Here, adaptive tracking of temporally autocorrelated risk should be possible if an individual produces mainly self-similar offspring, but not if parental phenotype has no effect on the distribution of offspring phenotypes. The evolutionary consequences of deterministic versus stochastic diversification and reproductive mode (sequential vs. clutch-based) on bet hedging under varying risk structures have yet to be rigorously investigated, and indeed, are a key area for future investigation.

Prior research has extensively characterized the fitness consequences of variation in stochastic switching rates using both theoretical and empirical tools (Thattai and van Oudenaarden 2004; Kussell and Leibler 2005; Leimar 2005; Wolf et al. 2005; Acar et al. 2008; Donaldson-Matasci et al. 2008; Salathe et al. 2009; Gaal et al. 2010; Visco et al. 2010; Libby and Rainey 2011). This work has examined the fitness consequences of phenotypic switching in ecological scenarios where the environment can take one of several distinct states. This ecological scenario, however, may be less applicable to macrobes than to microbes. Here we construct a simple model incorporating periodic disaster, the paradigmatic source of environmental fluctuation from studies of macrobial bet hedging (Cohen 1966; Clauss and Venable 2000; Simons 2009), separated into temporal and spatial components. This allows us to better understand the fitness consequences of diversification over a range of these ecological parameters. It should be noted that the temporal autocorrelation model of disaster events can be translated into standard distinct environmental state models by using a geometric distribution to calculate the waiting time until disaster events target a different phenotype. Nonetheless, temporal autocorrelation is but one axis of risk relevant to the fitness consequences of uncertainty—our model demonstrates that the spatial extent of risk also factors heavily into the fitness consequences of diversification rate.

Despite intensive research over the last 50 years, the microorganismal and macroorganismal bet-hedging literatures remain largely independent. Yet the underlying logic of the evolutionary theory of bet hedging applies to all organisms, big and small, and a robust understanding of bet hedging requires us to explain the differences between micro- and macroorganismal diversification strategies. In this article, we show that ecology can dramatically affect the evolution of diversification rate. Conditions favoring rapid diversification (broad spatial extent of disaster, no temporal autocorrelation in risk, small populations, limited migration among populations, and frequent disaster relative to generations) may often be faced by macroorganisms, but may rarely be encountered by microorganisms. Testing these predictions will require much more insight into the nature of risk in natural populations, especially among microbes. Microbial ecology has always been challenging to study, but recent advances in the field (e.g., high-throughput gene expression data from natural populations [Konopka and Wilkins 2012; Marchetti et al. 2012]) may allow fine-grained study of the spatial and temporal dynamics of disaster.

## Data Archiving

The doi for our data is 10.5061/dryad.370rv.

## Supporting Information

Figure S1 Stochastic switching, diversification rate, and a visual description of the model. **Figure S2:** Temporal autocorrelation of risk in a multicellular life history.
