# Supplementary material for: Courting disaster: How diversification rate affects fitness under risk
Source: Evolution. 2014 Dec 23;69(1):126–35. doi: 10.1111/evo.12568 (PMC4312886; doi:10.1111/evo.12568)
Supplement: Figure S1 — Stochastic switching, diversification rate, and a visual description of the model. Figure S2: Temporal autocorrelation of risk in a multicellular life history. [file evo0069-0126-sd1.docx]

**SUPPLEMENTARY FIGURES**

**
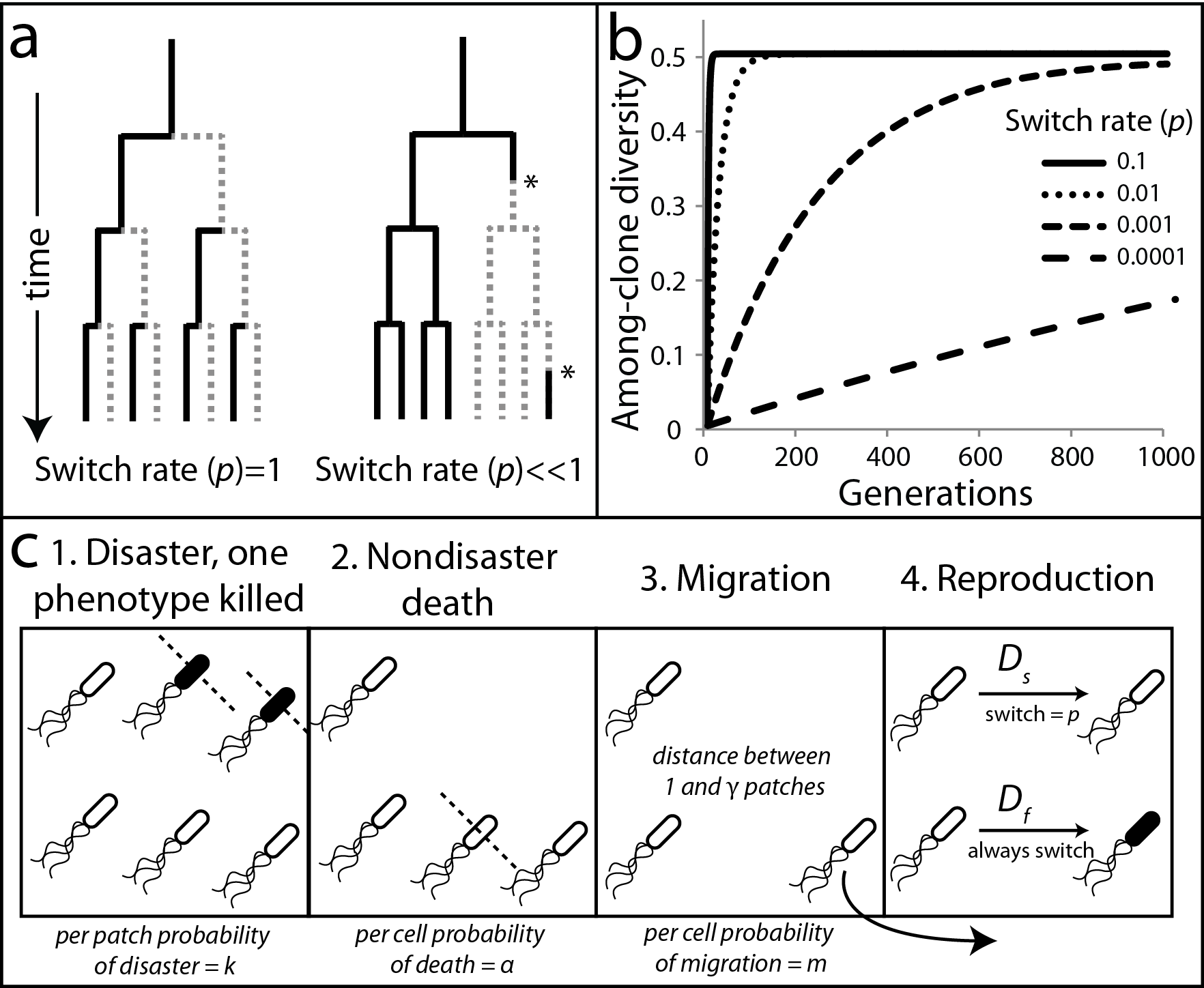
**

**Figure S1.** a) Simplified phylogeny illustrating the difference between fast and slow switching lineages. Black and gray lines represent an individual’s phenotype. With rapid (*p*=1) diversification, a single organism creates phenotypically-heterogeneous offspring each generation. In contrast, slow (*p*<<1*)* phenotype switching (denoted by asterisks) generates diversity among different lineages, but individuals rarely produce offspring of a different phenotype. b) Slow rates of switching require many generations to generate optimal diversity. At a typical switch rate of 10^-3^ / generation, it takes more than 1000 generations to generate a 50:50 ratio of A and B. c) Progression of events during a single time step of our stochastic metapopulation simulation model (see text for full description). All four panels depict the same patch during one time step.

Derivation of switching rate for Figure S1b

Consider a genotype that switches between two phenotypes, A and B. Assuming each phenotype is equally fit before the occurrence of some future environmental disaster, the rate of phenotypic differentiation is:

$\frac{dA}{dt}={-p}_{B}A+p_{A}(1-A)$ *(1)*

where A is the frequency of the A phenotype in the group, and *p_A_* is the probability of switching to the A phenotype and *p_B_* the probability of switching to the B phenotype. Solving for the frequency of the A phenotype after time in generations *t,* we obtain:

$A\left( t \right)=\frac{p_{A}}{p_{A}+p_{B}}+e^{-\left( p_{A}+p_{B} \right)t}C$ *(2)*

When the group is initially composed entirely of A phenotype individuals, the integration constant *C* = *p_B_* / (*p_a_* + *p_b_*). Using the diversity index Simpson's D (Simpson 1949), we can calculate patch-level diversity as:

$S_{D}\left( t \right)=1-(A\left( t \right)^{2}+(1-A{\left( t \right))}^{2})$ *(3)*

As Fig. S1b shows, a phenotypically uniform population can take hundreds of generations to re-establish a high degree of phenotypic diversity, even with relatively high switching probabilities.


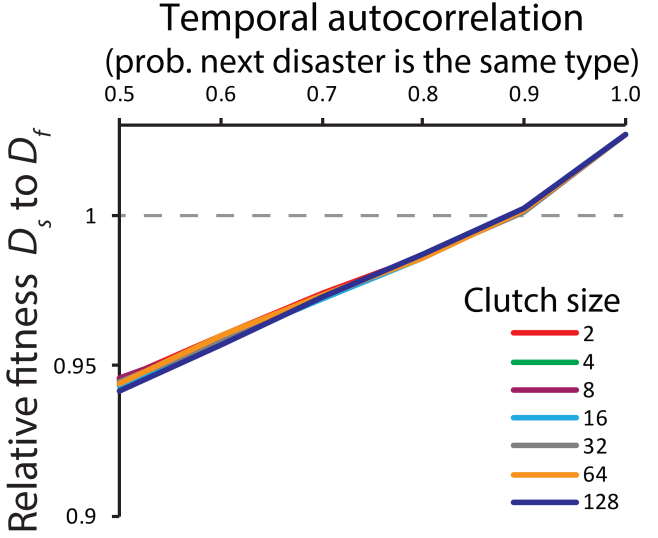


**Figure S2**- We modified our model to simulate a multicellular life history. As with the unicellular model, reproduction is constrained by the carrying capacity of the patch. We selected individuals at random from the patch to reproduce until the carrying capacity of the patch was met. *D_f_* produced 50% A and 50% B phenotype offspring, while *D_s_* produced offspring that each had a chance *p* of being phenotypically different from the parent. We find that our results were not affected by clutch size, or whether the model considered a uni- or multicellular life history (compare these results to the green dashed line in Fig. 2c, the unicellular version of this simulation). For all simulations *a*=0.1, *k*=0.1, *m*=0, *p*=0.001. Plotted for each parameter combination is the mean relative fitness of 1000 simulations of a 10 patch metapopulation, with each patch containing a maximum of 10^5^ individuals.
